# Pyruvate Dehydrogenase Complex Deficiency: A Review of Treatments and Case Series

**DOI:** 10.3390/ijms27062732

**Published:** 2026-03-17

**Authors:** Batya Betesh-Abay, Eilon Shany, Orna Staretz-Chacham, Ilan Shelef, Abed N. Azab

**Affiliations:** 1 Department of Nursing, School for Community Health Professions, Faculty of Health Sciences, Ben-Gurion University of the Negev, Beer-Sheva 8410501, Israel; tobibetesh@gmail.com; 2 Neonatal Department, Saban Children Hospital, Soroka University Medical Center, Beer-Sheva 8410101, Israel; eshany@bgu.ac.il (E.S.); staretz@bgu.ac.il (O.S.-C.); 3 Faculty of Health Sciences, Ben-Gurion University of the Negev, Beer-Sheva 8410501, Israel; 4 Rare Disease Center, Saban Children Hospital, Soroka University Medical Center, Beer-Sheva 8410101, Israel; 5 Radiology Department, Soroka University Medical Center, Beer-Sheva 8410101, Israel; 6 Department of Clinical Biochemistry and Pharmacology, Faculty of Health Sciences, Ben-Gurion University of the Negev, Beer-Sheva 8410501, Israel; 7 The School of Brain Sciences and Cognition, Ben-Gurion University of the Negev, Beer-Sheva 8410501, Israel

**Keywords:** pyruvate dehydrogenase complex deficiency, inborn error in metabolism, ketogenic diet, dichloroacetate

## Abstract

Pyruvate dehydrogenase complex deficiency (PDCD) is a heterogenous mitochondrial inborn error in carbohydrate oxidation manifesting as congenital lactic acidosis. PDCD presents diagnostic and therapeutic challenges. While no curative treatment exists for PDCD, certain therapeutic modalities may improve prognosis and ameliorate symptom severity. This article examines the effectiveness of treatments for PDCD and presents a case series of three patients with PDCD. A scoping literature review was conducted for treatments of PDCD. Patient data for case reports was extracted retrospectively from electronic medical records from a large tertiary hospital. We reviewed and summarized findings from seven preclinical studies and ten human studies, which showed that dichloroacetate and the ketogenic diet were the most frequently studied treatments. Therapeutic approaches observed select positive outcomes such as reduced lactate levels, improved neuropathological manifestations, and increased longevity. However, most interventions have yet to be rigorously investigated. Early diagnosis of PDCD is integral, as treatment methods may offer improved clinical and biochemical outcomes. Clinical trials of existing and novel treatments are necessary to improve management and further understand the prognostic potential of this metabolic disorder.

## 1. Introduction

The mammalian body subsists on three essential nutrients for survival: carbohydrates, lipids, and proteins. As each nutrient enters the body, corresponding metabolic pathways are triggered, breaking down these nutrients and converting them into usable energy, among other biological products [1]. Carbohydrate metabolism involves the transformation of glucose into adenosine triphosphate (ATP) through aerobic respiration via a series of intracellular cascades known as glycolysis and the tricarboxylic acid (TCA) cycle [1,2].

**Figure 1:**
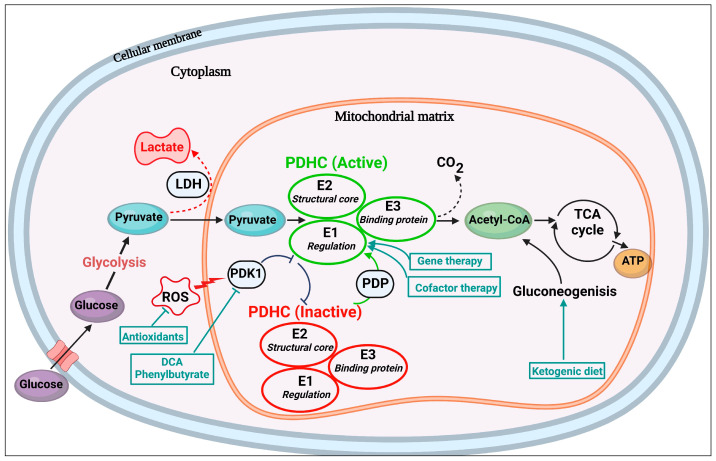
Activity and regulation of the PDC. The PDC is situated inside the cellular mitochondrial matrix. When enzymatic function is disrupted, pyruvate is reduced to lactate (via LDH) instead of acetyl-Coa. Therapeutic interventions for PDCD are indicated in teal. Inhibitory (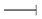) and facilitatory (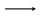) arrows are provided for indication. Abbreviations: ATP, adenosine triphosphate; CO_2_, carbon dioxide; DCA, dichloroacetate; LDH, lactate dehydrogenase; PDHC, pyruvate dehydrogenase complex; PDK, pyruvate dehydrogenase kinase; PDP, pyruvate dehydrogenase phosphatase; ROS, reactive oxygen species; TCA, tricarboxylic acid.

The pyruvate dehydrogenase complex (PDC) facilitates the transition from glycolysis to TCA, oxidizing pyruvate into acetyl-coenzyme-A (coA) [2,3]. Situated in the mitochondrial matrix, PDC is structurally composed of multiple copies of three subunits: (i) E1, containing alpha and beta components, a protein determinant regulating the active, unphosphorylated state of the complex; (ii) E2, forming the structural core; and (iii) E3, a binding protein [1,4,5] (see Figure 1 for illustration). Furthermore, PDC is contingent upon three cofactors for function: thiamine pyrophosphate, lipoic acid, and flavin adenine dinucleotide [5].

Congenital PDC dysfunction hinders mitochondrial oxidation and results in pyruvate reduction to lactate instead of acetyl-coA [6]. Lactate produces only a small fraction of ATP, and when present in the body at unregulated capacities can lead to irreversible tissue damage by means of hypoxia [7]. Given the explanation above, PDC deficiency (PDCD) manifests as congenital lactic acidemia, and is generally accompanied by varying neuropathological symptoms, given that optimal brain function is highly reliant on the aerobic oxidation of glucose [6,8].

Encoded by nuclear genes, PDC mutations are most frequently (76–85%) located on the E1 alpha subunit-encoding allele (PDHA1), and thus most of the hereafter described features apply to this form of the illness [9,10]. Diagnosis of PDCD can be made through low enzyme activity in cultured fibroblasts, lymphocytes, and/or skeletal muscle as well as genetic sequencing [6,8]. Other common but inconclusive and non-specific testing include cerebral spinal fluid (CSF) examination, urine analysis and blood tests for measurement of lactate (and lactate to pyruvate ratios) [6,11], proton magnetic resonance spectroscopy of the brain for detection of nervous system lactate buildup [8], and magnetic resonance imaging (MRI) for brain abnormalities [12,13,14]. In the largest multicenter cohort study to date, 891 patients with PDHA1 were analyzed (53% females) [9]. Genetic analysis identified 331 distinct PDHA1 variants, predominantly missense (50%), followed by frameshift (20%), with the majority (69%) being private. Most variants were reported as *de novo* (75%), whereas maternally inherited variants were more frequent among males [9]. Mutational hotspots were observed in exons 5–11, with several variants exhibiting sex-specific enrichment. The median age of onset was 4 months, with females presenting earlier than males, and neonatal or infantile presentation associated with more severe outcomes. Notably, 47% of the cases exhibited fetal or perinatal abnormalities, including intrauterine growth restriction (IUGR) or microcephaly [9]. The majority of patients exhibited developmental delay, intellectual disability, hypotonia, abnormal movements, seizures, feeding difficulties, and microcephaly [9]. Neuroimaging commonly revealed basal ganglia involvement (39%), cerebral atrophy (38%), and corpus callosum anomalies (27%), while non-neurological features included dysmorphic facial traits, skeletal abnormalities, and osteopenia [9]. Residual PDC enzyme activity can vary across tissues (median 31–36%), reflecting marked heterogeneity. Survival analysis shows a median survival of 13 years, with females surviving significantly longer than males; male sex, neonatal presentation, and select variants may increase the risk of early mortality. Overall, genotype, sex, and age at presentation strongly correlate with phenotypic severity, emphasizing the heterogeneous clinical spectrum of PDHA1-related PDCD [9].

While PDCD is a rare inborn error in carbohydrate metabolism, it is among the leading causes of congenital lactic acidosis (CLA) according to the North American Mitochondrial Disease Consortium Registry [15] and among the prevalent disorders of mitochondrial metabolism [6,15,16,17,18]. Due to its scarcity, clinical unfamiliarity, and diagnostic challenges, there are conceivably many more cases than the nearly 1000 documented in the literature (predominantly of mutations of the E1 alpha subunit) [9]. PDCD is clinically devastating and should be highly considered among all patients with CLA and early onset neuropathy. Further research of PDCD is necessary in order to enrich clinical awareness and optimize management strategies.

This review aims to examine the effectiveness of the current modalities for the treatment of PDCD, and to present an illustrative case series of three patients with PDCD demonstrating the variability in presentation, treatment, and outcomes.

## 2. Treatments

At present, no curative treatment exists for PDCD [11]. Due to the ambiguous presentation of the illness and the challenges in obtaining an official and timely diagnostic confirmation of PDCD, treatment prior to diagnosis is empirical and can result in irreversible damage if initial inclinations are incorrect. For example, an infant with PDCD may clinically present similarly, or even identically, to one with an error in pyruvate carboxylase metabolism [6,16]. In pyruvate carboxylase deficiency, the gluconeogenesis pathway is impaired [19], and treatment conventionally involves frequent carbohydrate loading and stringent avoidance of glucose fasting [6]; contrastingly, in PDCD, this dietetic regimen can cause fatal outcomes, severely aggravating the malfunctional enzymatic complex and ensuing excessive lactate buildup [6,16,20]. Therefore, keen follow-up tracking response to treatment should be diligently practiced.

Therapeutic models for PDCD generally target two main mechanisms (see Figure 1): (i) avoidance of glycolysis activation—a pathway triggered by carbohydrate consumption (i.e., by administering the ketogenic diet) [21,22] and (ii) optimization of the PDC regulatory mechanisms and stimulation of residual unphosphorylated PDC activity (i.e., by administering cofactor therapy [relevant for select patient subgroups], dichloroacetate [dependent on residual enzymatic activity], and phenylbutyrate). Therapeutic interventions for accompanied symptoms are standard protocol as well (i.e., bicarbonate, anticonvulsants, and L-carnitine [for KD-induced secondary deficiency], among others) [11,23,24].

### 2.1. Ketogenic Diet

The ketogenic diet (KD) refers to a dietary regimen of ingestion of fats and proteins, relative to low carbohydrate intake [25]. Introduced in 1921 [26], its discovery was tailored to create a sustainable, ketone-producing diet in patients with diabetes and epilepsy, bearing metabolic resemblance to a state of fasting [25]. KD composition may vary from as low as a 1:1 fat-to-carbohydrate-plus-protein ratio, to as high as a 4:1 ratio. The justification for KD practice in PDCD offers bypassing glycolysis, and thus PDC activation, leaving the defective enzymatic complex ‘dormant’ and eliciting energy production from alternative metabolic pathways [22], rendering the KD as the classic base therapy for PDCD [11]. Nonetheless, ketone energy production is not synonymous with glucose energy production given the acidic nature of ketones and the inability of all organs to convert ketone bodies to ATP (i.e., the liver [27]), among other shortcomings.

### 2.2. Dichloroacetate

Dichloroacetate (DCA) is a synthetic ionic compound structurally analogous to pyruvate [28]. DCA inhibits the four isoforms of pyruvate dehydrogenase kinase (PDK) [29]. PDK-1 in particular downregulates PDC activity, depleting pyruvate oxidation and leading to increased pyruvate-to-lactate conversion [30]. The pharmacological revelation of DCA was first documented in 1973 [31]. Kinetically, DCA operates by inhibiting PDK activity, leading to reduced phosphorylation of the PDC E1 alpha subunit; this maintains the residual dephosphorylated and catalytically active state of PDC [32,33], in turn reducing serum lactate levels at their source [34].

### 2.3. Phenylbutyrate

Sodium phenylbutyrate is an ammonia scavenger prodrug, primarily indicated among patients with urea cycle disorders for the prevention of hyperammonemia [35]. The drug metabolizes to phenylacetate and subsequently conjugates with glutamine, becoming phenylacetylglutamine. This reaction allows for the circumvention of microphysiological pathways, opening an alternative route for nitrogen waste excretion in patients with select defective enzymes [36]. Phenylbutyrate has been observed to inhibit PDK 1, 2, and 3, thus enhancing PDC activity [37]. Furthermore, persistent hyperammonemia has been reported among patients with PDCD, suggestive of another potential benefit of phenylbutyrate treatment [38,39,40].

### 2.4. Triheptanoin

Triheptanoin is a medium-chain triglyceride composed of three 7-carbon fatty acids. Triheptanoin is rapidly metabolized in the gut, forming molecules of glycerol and heptanoate. Heptanoate is an odd 7-carbon fatty acid whose oxidation may derive both from acetyl-coA and propionyl-CoA. Unlike even-chain fatty acids which are oxidized to acetyl-coA only, heptanoate initially yields propionyl-CoA that is subsequently converted to succinyl-CoA, a TCA cycle intermediate. Therefore, metabolism of triheptanoin can induce two key substrates for the TCA cycle: acetyl-CoA and succinyl-CoA [41]. Thus, it is hypothesized that triheptanoin may serve as an alternative energy source in PDCD, in contrast to the even-chain fatty acids produced from ketosis [42].

### 2.5. N-Acetylcysteine

N-acetylcysteine (NAC) is an antioxidant and mucolytic agent indicated in a variety of respiratory and other conditions [43]. The pharmacological aspect that suggests NAC use in mitochondrial diseases is related to its reactive oxygen species (ROS)-reducing ability and subsequent increased ATP production during cellular oxidative stress (i.e., PDCD). The kinetic cascade that augments this process is NAC’s role as a precursor to cysteine and influencer in glutathione synthesis [44]. Pathophysiological mechanisms of PDC variants show enhanced ROS generation; thus, antioxidant administration may serve as a beneficial therapeutic approach [11,45].

### 2.6. Cofactor Therapy

Cofactors (coenzymes and vitamins) are essential to enzymatic function, obliging consumption from an external source. This phenomenon is exemplified in the case of beriberi [46] and Wernicke’s encephalopathy [47] (i.e., secondary PDC deficiency [11]), where patients with no congenital metabolic condition exhibit obstructed energy production and neuropathological manifestations in a state of vitamin B1 (thiamine) deprivation [48]. The rationale for cofactor therapy in PDCD stems from the reactive dynamics between thiamine/lipoic acid and the E1 alpha subunit of PDC [49,50], which regulates enzyme activity and undergoes dephosphorylation [5] and which harbors the most common PDC genetic defects [10]. Notably, the beneficial effects of thiamine are reported among a minority of patients, particularly those with mutations in exon 3 or the thiamine pyrophosphate binding site of the E1 alpha subunit. Additionally, alpha lipoic acid and flavins (such as flavin adenine dinucleotide) possess ROS-attenuating properties [51], and therefore may theoretically bear therapeutic relevance, in similarity to NAC [45].

### 2.7. Gene Therapy

While genetic disorders were once perceived as incurable, treating such diseases by means of gene therapy has recently demonstrated success in select defects (i.e., adenosine deaminase deficiency and X-linked severe immunodeficiency disease) [52]. The PDC is encoded exclusively by nuclear genes. This conclusive piece of data encouraged researchers to investigate a recombinant adeno-associated virus (rAAV) vector containing a protein construct designed to modify select mutations of PDCD in an attempt to amend the error in genetic makeup that leads to PDCD, at least in part [53,54].

## 3. Treatment Outcomes

### 3.1. Data Sources and Study Selection

This review was designed by the authors and was not conducted in accordance with the guidelines of Preferred Reporting Items for Systematic Reviews and Meta-Analyses (PRISMA) [55]. We did not perform a systematic review because the literature on the subject is highly limited. Adherence to PRISMA guidelines would have required the exclusion of landmark studies due to, for example, bias in study cohort selection [56] and patient overlap across studies [57]. The databases searched included: Cochrane Central Register of Controlled Trials, PubMed, and Google Scholar, customized to the following years: 1990–2025. In the search field we used the keywords: *Pyruvate dehydrogenase deficiency* individually, and in combination with each of the following words: *treatment/s*, *efficacy*, *clinical trial/s*, *randomized controlled trial*, *ketogenic diet*, *vitamin therapy*, *cofactor therapy*, *thiamine*, *lipoic acid*, *flavin adenine dinucleotide*, *phenylbutyrate*, *triheptanoin*, *dichloroacetate*, *n-acetylcysteine*, *antioxidant*, and *gene therapy.* This review included both preclinical and human studies that examined outcomes of therapeutic interventions for PDCD. To qualify for inclusion, patient cohorts were required to have a confirmed diagnosis of PDCD. For preclinical studies, only model samples with a verified PDC defect were included. All included works had to evaluate at least one of the following measured outcomes, as relevant to study design: a biochemical response, (including: lactate to pyruvate ratio, blood/urine/CSF lactate levels, or PDC enzymatic activity or immunoreactivity), or a clinical response, (including: neurological or non-neurological symptomatic manifestations, neuroimaging changes, illness exacerbation, hospitalization time, patient development/physical state/quality of life/functionality/growth, or mortality). Case reports were excluded, however case series of ≥3 patients were eligible for inclusion. Findings were limited to those published in English. The authors discretionarily included studies that investigated treatments for CLA on the premise that the following two criteria were upheld: (i) an appreciable portion of the referenced study cohort sustained a diagnosis of PDCD and (ii) the intervention directly influences the kinetic behavior of the PDC. This methodical decision was based on the fundamental understanding that, though phenotypically heterogenous, all cases of CLA derive from hindered mitochondrial energetics and malfunctional pyruvate oxidation (i.e., lactate is a molecular derivative of pyruvate) [7,29], as well as the challenges faced in conducting trials of such a rare disease.

Only publications in peer-reviewed journals were considered for eligibility. The authors (A.N.A and B.B-A) reviewed the titles and abstracts of all discovered articles and discerned relevance.

### 3.2. Reported Effectiveness of Treatments for PDCD

We report the findings of 17 eligible publications; 10 human studies are tabulated in Table 1 and seven preclinical studies in Table 2. No published findings of randomized clinical trials (RCTs) of an exclusive PDCD population were found in our search. However, four studies of CLA were included, three examining DCA [29,58,59] and one on thiamine [56], two of which were RCTs [58,59]. Table 1 presents 170 patients with PDCD. Regarding the location of subunit mutations of the populations studied, 45 were not specified, 107 were of the E1 unit (65 of which were of E1 alpha, one of which was E1 beta, and 41 were not specified [alpha or beta]), and 18 had mutations of the E3 subunit. Among these, 84 patients were included in the DCA studies [29,34,58,59], 60 in KD [21,57,60,61], and 26 with thiamine therapy [56,62]. Patient-overlap across studies was reported [57]. No further human studies examining alternative treatment methods were uncovered in accordance with our search criteria. Table 2 displays seven preclinical studies investigating in vivo or in vitro treatments for PDCD including: two studies on DCA [32,63], one on KD [64], one on phenylbutyrate [65], one on gene therapy [53], and two investigating combination treatments of DCA with phenylbutyrate [65] and gene therapy with DCA [66].

All included studies reported select positive outcomes, as pertinent to intervention modality and study design. Below, the effects of treatments are summarized, categorized by treatment method and response type, as depicted in Table 1 and Table 2.

#### 3.2.1. DCA

*Biochemical response*: A marked reduction in blood or CSF lactate concentrations was reported with use of DCA among all human studies [29,34,58,59], at varying degrees of intensity and conditions (meal-induced vs. basal), as interconnected with study design, cohort size and analysis approach. Importantly, the benefits were only noted among those with residual enzymatic activity. Among preclinical studies, an increase in maximal PDC enzymatic activity was found in in vitro fibroblast human cell lines following the treatment of DCA [32,63].

*Clinical response*: Findings reported improved neuropathic trends [29,34,58] and suggested reduced mortality [59] among patients administered DCA therapy. Contrastingly, an observed association between DCA and select neuropathological negative outcomes was documented by Stacpoole et al. [59]. In this context, we note that although the current review does not assess safety/tolerability of treatments, DCA has been suggested to have neurotoxic properties [67] and the clinical benefits and adverse effects of DCA are therefore intricately linked. Nevertheless, Stacpoole et al. regarded this negative effect to plausibly be attributed to the natural progression of the disease and not necessarily pertinent to DCA treatment.

#### 3.2.2. KD

*Biochemical response*: Among the four human studies researching KD practice in PDCD, two reported reduced blood lactate levels [21,60]. The other two [57,61] did not report such findings. In the preclinical study [64] applying the KD on a zebrafish model, reduced lactic acidosis was documented.

*Clinical response:* Notable clinical outcomes were documented in the KD studies: Wexler et al. [60] reported KD associating with increased longevity and improved mental development. Weber et al. [57] found the KD to correlate with improved neurodevelopment. Staretz-Chacham et al. [61] observed that the KD improved patient survival and reduced hospitalizations; however, no improvement in quality of life or neuroimaging were noted and raised the possibility that a more restrictive KD may be more beneficial. Additionally, positive effects on epilepsy, ataxia, sleep disturbance, speech and language development, social functioning, and frequency of hospitalizations were documented by Sofou et al. [21]. In the preclinical KD study [64], restored vision, improved feeding behavior, and increased survival were reported.

#### 3.2.3. Thiamine

*Biochemical response:* Increased PDC activity and reduced blood and CSF lactate concentrations were noted by Naito et al. [56] when high-dose thiamine was administered among select genotypes of PDCD.

*Clinical response:* Using descriptive analysis, Dongen et al. [62] found thiamine therapy to resolve brain lesions and improve neuropsychological performance, mobility, respiration, strength, energy levels, development, intellectual abilities, and communication among select patients in their study cohort. In the other study [56] examining thiamine treatment, improved hypotonia, ataxia, paralysis, respiration, vomiting, and muscle pain was reported.

#### 3.2.4. Phenylbutyrate

*Biochemical response:* Ferriero et al. [65] documented increased unphosphorylated PDC-E1 alpha and PDC enzyme activity in mouse brain, muscle, and liver in in vitro laboratory models.

#### 3.2.5. Combination Therapies

*Biochemical response*: Han et al. [66] investigated gene therapy rAAV vectors while concurrently treating in vitro models with DCA. Their findings showed increased E1 alpha expression of 40–60%; they concluded that DCA increased PDC activity by up to 90%, thereby improving the expression of E1 alpha proteins. An additional study [65] exploring concomitant in vivo and in vitro administration of phenylbutyrate and DCA found a greater increase in PDC activity compared to each drug alone.

## 4. Case Series

Hereafter, we present a three-part case series of patients with PDCD. The cases are included as descriptive clinical vignettes intended to complement the review. While these three cases are not intended to represent the full phenotypic spectrum of PDCD, they exemplify the disorder’s inherent heterogeneity. The patients differ substantially in onset, clinical presentation, imaging findings, treatment strategies, and outcomes, exemplifying the variability described in the literature and reinforcing the key points of this review.

Data for case vignettes was extracted retrospectively from electronic medical records at Soroka University Medical Center (SUMC), Beer-Sheva, Israel, for patients with a diagnosis of PDCD, as genetically confirmed by a pathological variant in a PDC component enzyme or subunit. Publication of the case series received ethical approval from the Ethics (Helsinki) Committee in SUMC, waiving the need for informed consent. The case series examines three patients with distinct clinical presentations and therapeutic approaches, broadening the discussion of appropriate treatment strategies.

### 4.1. Case A

**Figure 2:**
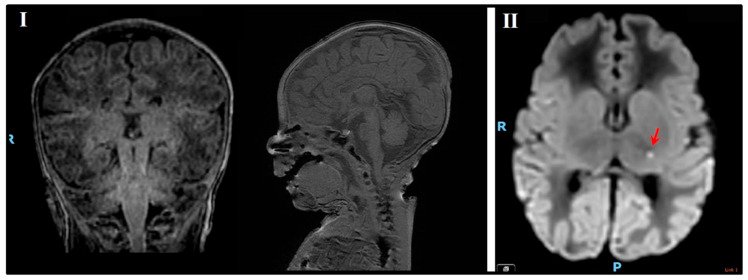
Postmortem MRI brain scan of male infant described in Case A. (**I**) T1 weighted imaging displaying absence of neuroanatomic defects. (**II**) Diffusion weighted imaging showing mild ventricular bleed, a thalamic infarct (Lt small) located in the left internal capsule as indicated by the red arrow. R = Right; P = Posterior.

A male neonate was born at 37 weeks’ gestation as a second child to nonconsanguineous parents weighing 2170 g (seventh percentile) with a head circumference of 33 cm (30th percentile). Birth was induced due to IUGR and preeclampsia of the mother. Apgar scores were 9 and 10, at one and five minutes, respectively. At two weeks old, the neonate was brought to the emergency department for abnormal breathing, as reported by the parents. At triage, vital signs were within the normal range and body weight was 2145 g. Venous blood was drawn, ruling out indicators of infection/sepsis. Liver transaminases were eminently elevated (gamma-glutamyl transferase 794 U/L and alkaline phosphatase 777 U/L). After a pediatrician’s examination, the infant was discharged, and the parents were instructed to carefully monitor weight gain. At one month old, the infant was brought once again to the emergency department with complaints that he went blue and floppy, perspired, and was feeding poorly. Upon examination he was severely hypothermic (32.6 °C), hypotonic, and sustained an incarcerated inguinal hernia. A venous blood gas test revealed the following: pH = 6.94, pCO_2_ = 43 mmHg, pO_2_ = 53 mmHg, HCO_3_^−^ = 9.2 mmol/L, and lactate = 9.02 mmol/L (normal ranges: pH: 7.35–7.45, pCO_2_: 35–45 mmHg, pO_2_ = 69–116 mmHg, HCO_3_^−^: 22–26 mmol/L, lactate: 0.5–2.2 mmol/L). The patient was admitted to the pediatric intensive care unit where he underwent further testing, finding no underpinning etiology for his state (no detectable hypoxic source point). Consultation with a metabolic specialist inferred differential diagnoses of PDCD, pyruvate decarboxylase deficiency, or other mitochondrial disorders. Breast milk consumption was halted, and the infant began a continuous intravenous infusion of dextrose 10% sodium chloride 0.33%. The following pharmacological regimen was started: intravenous ceftriaxone, arginine injection, dopamine infusion, vitamin B_7_, bicarbonate sodium, carnitine injection, thiamine, riboflavin, and vitamin B12. The next day the infant’s serum ammonia began to rise (221.7 ug/dL); treatment of 4-phenylbutyrate sodium injection and sodium benzoate solution was added. The patient deteriorated as seen in arterial blood gas trends displayed in Table 3. On day three of hospitalization, he died with ammonia levels elevated exponentially to 1170.9 ug/dL and lactate 24.8 mmol/L (Table 3). Postmortem MRI displayed no anatomical brain abnormalities (Figure 2I), mild ventricular bleed, a thalamic infarct (Figure 2II) and ureteral and vesical distention. Autopsy results demonstrated adequate morphological appearance of internal organs with no evidence of malformation. Whole exome sangar sequencing was performed by Centogene, Italy, confirming a de novo missense mutation at the PDHA1 variant c. 763C>A p.(Pro255Thr), findings consistent with a diagnosis of pyruvate dehydrogenase E1 alpha deficiency.

### 4.2. Case B

**Figure 3:**
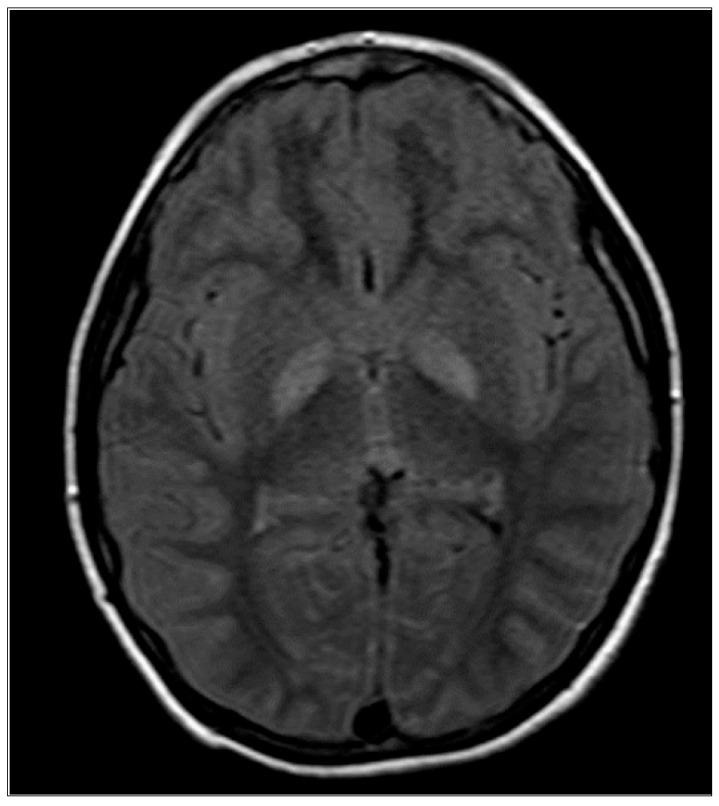
MRI brain scan of Case B. FLAIR sequencing found in MRI of Case B at eight years of age.

This patient is a 14-year-old male, born as a third child to non-related parents. His siblings are healthy, and no family history of genetic disorders is known. Gestation, birth, infancy, and toddlerhood were uneventful. Growth followed trends of normal range. Feeding difficulties were not noted. At three years of age, he was presented to the hospital with severe exercise-induced dystonia. Trihexyphenidyl therapy was administered, rendering minimal improvement. No etiology for dystonia was reported. At the age of seven years, he presented to the emergency department with a streptococcal throat infection in which the patient deteriorated to an apathetic state and was unable to move his extremities or speak. Elevated lactate was found in CSF testing (7.3 mmol/L). Mitochondrial disorder was suspected. Subsequent sequencing confirmed a mutation within exon 7 of PDHA1 hemizygous c.376>T, causing the substitution of arginine 126 with cystine. At eight years his dystonia severely exacerbated. An MRI scan displayed a fluid-attenuated inversion recovery (FLAIR) sequence revealing an abnormal signal in the globus pallidum with signs of restriction of the diffusion-weighted imaging (DWI) sequence, as seen in Figure 3. KD therapy was commenced. To date (age 14 years), he has persistent dystonia in his left hand, bears an asymmetric smile, nystagmus, and walks on his tippy toes with a limp. Additionally, his mitochondrial dystonia has severely exacerbated, causing distortion and full dysfunction of his left arm; he sustained a femoral fracture attributed to his hindered mobility. His therapeutic regimen includes KD, trihexyphenidyl, clobazam, L-carnitine, tetrabenazine, pyridoxine, vitamin B12, and thiamine.

### 4.3. Case C

**Figure 4:**
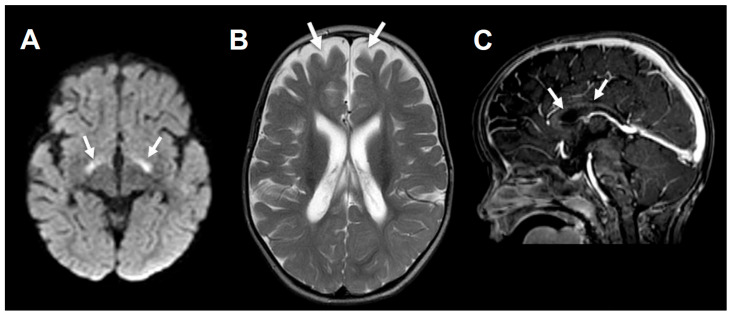
MRI brain scan of the female presented in Case C. At age one year the images display: (**A**)—diffusion-weighted imaging: bilateral restriction in the globus pallidus (arrows); (**B**)—T1-weighted imaging with gadolinium: thinned corpus callosum (arrows); (**C**)—T2-weighted imaging: moderate ventricular enlargement of the extraxial space (arrows) with mild ventricular dilatation.

**Figure 5:**
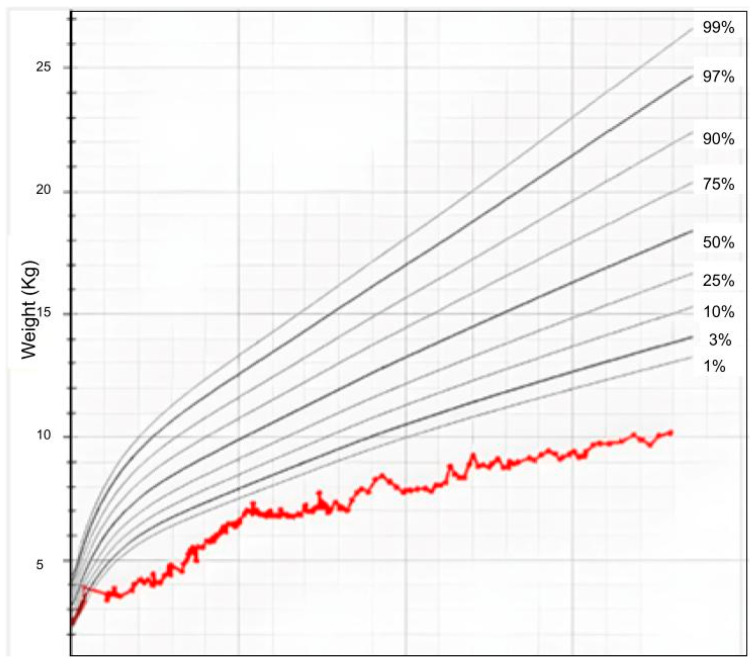
Growth chart of the female presented in Case C. Growth trend (red curve) for weight in accordance with the World Health Organization curve.

Patient *C* is a five-year old female and the second child born to consanguineous parents, carrying a known perpetuated familial mutation within the E3 subunit of dihydrolipoamide dehydrogenase (homozygous D479V NM–000108.5), as described previously [61,68]. She was born by normal delivery at 40 weeks. Two previous siblings demised at three days of age due to PDC-E3 deficiency. The patient’s birthweight was 2480 g (seventh percentile), with a head circumference of 31 cm (eighth percentile). Her Apgar scores were 9 and 10 at one and five minutes respectively. At a few hours of age, she was hypoglycemic with severe lactic acidosis on venous blood gases (pH: 6.9, lactate: 15 mmol/L, bicarbonate: 5 mmol/L). Supplemental intravenous sodium bicarbonate, dextrose 5%, and intralipid infusions were administered. Percutaneous endoscopic gastronomy (PEG) was performed in order to commence neonatal KD therapy. Following an extended stay in the neonatal intensive care, the patient stabilized and was able to go home under her parents’ care. An MRI scan from the age of one year displayed bilateral restriction in diffusion-weighted imaging in the globus pallidus, a thinned corpus callosum, moderate enlargement of the extraxial space with ventricular dilatation (Figure 4). She regularly visits the metabolic clinic at Soroka University Medical center (SUMC) for follow-up, with periodic exacerbations of metabolic crisis of lactic acidosis requiring hospitalization. At five years, she weighs 10.20 kg and 90 cm in height, markedly below the expected growth curve, as illustrated in Figure 5. She is severely developmentally disabled; she can sit with assistance, turns over, and attempts standing position. She is nonverbal and makes incoherent sounds. She will occasionally eat orally but frequently refuses. She mostly intakes formula of KetoCal (Nutricia, Gaithersburg, MD, USA) combined with regular formula via PEG. She receives the following daily medications: sodium bicarbonate, potassium citrate, and levetiracetam.

To facilitate comparison, Table 4 presents a summary of the three patients presented, including genotype, age at onset, major clinical features, treatments, and outcomes.

## 5. Discussion

This study reviewed the literature for the most established treatment methods investigated thus far for PDCD. While 17 studies met the eligibility criteria, none were RCTs of exclusively PDCD patients. Though scarce, collective research found select positive biochemical and clinical outcomes associated with KD, DCA, thiamine, and phenylbutyrate. Our findings reinforce the potential lactate-reducing mechanisms of DCA, with several studies reporting reduced blood and CSF lactate levels upon use. The KD practice improved symptomatic patient presentation, including increased lifespan and ameliorated neuropathology in some of the patients. The preclinical investigation of gene therapy proposed promising progressive findings in improving PDC enzymatic functionality. Nonetheless, no available treatment is ideal, and the trajectory and prognosis of PDCD remains poor.

The lack of RCTs emphasizes the inherent challenges underpinning furthering the research on this rare illness. To this end, we note that during our search process we found four ongoing clinical trials partially relevant to our review: a pivotal phase 3 randomized, double-blind, placebo-controlled trial [69] has enrolled 24 evaluable participants with genetically confirmed PDCD to evaluate the efficacy and safety of DCA through a caregiver-reported functional outcome measure, genotype-based dosing, and an open-label extension phase; recruitment is ongoing. A prospective long-term observational study [70] has enrolled 30 participants with epilepsy, GLUT1 deficiency, or PDCD to assess the metabolic and cardiovascular safety of KD therapy using carotid ultrasonography and biochemical monitoring; follow-up is expected to conclude in late 2025. A phase 2 pilot study [71] investigating a four-week course of sodium phenylbutyrate in a single PDCD patient focused on biochemical endpoints (blood lactate and pyruvate) and has been completed. Lastly, an open-label, proof-of-concept trial [72] is evaluating triheptanoin supplementation as an anaplerotic substrate in PDCD over a 2-year follow-up period, with active recruitment and interim metabolic assessments ongoing.

Among emerging therapeutic strategies for PDCD, triheptanoin, a medium-chain triglyceride originally developed for the treatment of long-chain fatty acid oxidation disorders, is currently under investigation in an open-label trial (as mentioned above) as an anaplerotic substrate therapy aimed at supporting oxidative metabolism in PDCD. Additionally, advancements in gene therapy offer a promising future direction for the management of mitochondrial disorders [73], by enabling targeted molecular correction of the underlying gene defects. These evolving strategies may lead to disease-modifying interventions for PDCD.

Our review excluded case reports/series of less than three patients, given that we were looking to amalgamate the most evidence-based treatment outcomes for PDCD and avoid anecdotal findings. Nevertheless, we maintain awareness that, in rare diseases, small study group analysis is a viable option. During data collection, we found two case reports/series that, although they did not meet inclusion criteria, stood out to us as scientifically and clinically influential; Inui et al. [74] novelly investigated the administration of *intravenous* KD on two female neonates sustaining PDC E1 alpha mutations. Among both patients, lactic acidosis improved upon KD commencement; neither patient displayed epileptic symptoms, and developmental outcomes were markedly bettered. Additionally, in a case–control design, Naito et al. [75] analyzed two patients (aged 10 months and 8 years) with PDCD treated on high-dose thiamine. Outcomes displayed improved blood lactate and pyruvate concentrations, as well as amelioration of certain clinical symptoms.

It is intriguing to examine the sex-related aspects of PDCD, specifically in respect to E1 alpha mutations, given that it is the most genetically common form of the illness [8,76]. Inheritance of the PDHA1 gene is etiologically X-linked dominant. Collectively, among females the illness has been reported as heterozygous, in contrast to hemizygous in males. Nonetheless, females may exhibit a recognizable and more severe clinical phenotype characterized by dysmorphic features and microcephaly. Furthermore, significant psychomotor delay and brain abnormalities (atrophy in the cortical and or subcortical regions, ventricular dilation, cysts, and absence of the corpus callosum) have been documented [77,78,79,80]. Contrastingly, other studies observed the presentation among males to be poorer with severe lactic acidosis, and higher lethality [76,81]. Discussions around this incongruity lean towards the concept of X-inactivation and the nature of the mutation itself establishing to what extent cells express the faulty E1 alpha subunit [76,82]. Across epidemiological studies, several mutations have been identified with codon patterns that are more pronounced in males than in females (R72, R263, and R378 making up nearly half of those carrying missense or nonsense mutations leading to protein misfolding). Conversely, females display markedly more insertion or deletion mutations. Despite these differences in characteristics, the illness occurs overall evenly across both sexes [82]. Recent large-scale cohort data demonstrated sex-specific disease expression particularly among X-linked PDHA1 deficiency [9]. In a cohort of 891 patients, females presented significantly earlier than males, with a median age of 2 versus 8 months, and de novo variants were reported more frequently in females (87%) than in males (63.7%), whereas inherited variants were more common in males (36% vs. 13%) [9]. Survival outcomes also differed markedly by sex: median survival was 17 years for females compared with 6 years for males, with males experiencing earlier mortality, particularly in cases of neonatal onset [9]. Importantly, variant type distribution showed sex bias, with missense variants more prevalent in males (76%), while frameshift/nonsense variants were significantly more frequent in females (33%), including enrichment of loss-of-function variants in nonsense-mediated decay–predicted regions. Moreover, female sex was associated with increased odds of developmental delay, seizures, microcephaly, dysmorphic features, cerebral atrophy, corpus callosum malformations, and ventriculomegaly, whereas male sex was associated with increased odds of peripheral neuropathy and abnormal basal ganglia findings [9]. Although elevated lactate was common across the cohort (90% in serum and 94% in CSF), systematic sex-stratified biochemical severity measures were not available [9]. Collectively, these findings underscore the importance of X-linked inheritance, X-inactivation, and mutation class in shaping phenotypic severity and prognosis. Our case series aligns partially with reported sex-linked variability. In *Cases A* and *B*, both males with PDHA1 mutations displayed markedly different phenotypic severity, ranging from neonatal fatality to chronic dystonia, reflecting the broad male clinical spectrum described in the literature. Conversely, the female patient (*Case C*) with DLD presented with profound developmental impairment; in DLD, severity pertains namely to the phenotype wherein sex is of no relevance [9,68].

In comparing DCA to KD, no direct comparative RCT has been conducted; such a design would seemingly not be feasible given the rarity and heterogeneity of PDCD. Importantly, according to the *Mitochondrial Disease Foundation* [83], the KD is listed as the first-line treatment for PDCD, while oral DCA was denied approval by the FDA as a treatment for PDCD in September 2025, due to insufficient evidence for efficacy. Notably, in the DCA trials, the medication was often administered as an adjunct to diet, as described in the study plan [69]. Reported outcomes with DCA vary considerably across studies as seen in Table 1 and Table 2, reflecting differences in residual enzymatic activity, treatment duration and heterogeneity of the illness. Of relevance, DCA kinetically works through the mechanism of increasing both the catalytic activity and stability of the PDC enzymatic complex, whereas the KD is applied in order to avoid enzymatic activation altogether (see Figure 1 for illustration).

We present a case series of patients with PDCD. Our case series was conducted in a single medical center, which may lead to selection bias and limit generalizability. Our hospital serves a regional population including a substantial Arab-Bedouin (Muslim) community, where consanguineous marriage patterns contribute to a high prevalence of the E3 (DLD) subunit mutation, as described previously [61,68]. Although this mutation represents one of the rarest variants of PDCD subtypes reported globally, we intentionally avoided presenting only such cases, as this would have limited the broader relevance of our findings. Instead, we deliberately selected patients with heterogeneous genetic and clinical presentations to better illustrate the variability of PDCD manifestations and therapeutic responses. This approach, while more biased, offers more generalizability in an epidemiological sense, attempting to depict the phenotypic spectrum of the disease to the best of our ability, based on the available cohort.

Furthermore, each case provides a novel contribution to the relevant literature. In particular, *Case A* exhibited no anatomical brain abnormalities on imaging yet experienced a rapidly progressive and fatal clinical course, underscoring the unpredictability of disease severity and treatment response. *Case B* harbored a similar variant to Case A; however, in contrast, his illness manifested significantly later in childhood, triggered by infection. Additionally, *Case B* demonstrated a distinctive FLAIR MRI pattern that is less commonly described in PDCD. *Case C* represents a patient from the local Arab-Bedouin population, contributing to the limited reports of the rarest form of PDCD—DLD—globally. Generally speaking, genetic variation among PDCD subtypes influences both disease severity and therapeutic response. The E1 alpha (PDHA1) gene is most frequently affected [10] encompassing a wide phenotypic spectrum, from severe neonatal presentations in male infants with possibly no residual enzymatic activity (often resulting in fatal lactic acidosis or Leigh-like encephalopathy), to more treatable forms that preserve partial enzymatic function, particularly those involving the thiamine pyrophosphate binding site. These patients may respond to high-dose thiamine and exhibit metabolic stabilization with a KD. However, the specific location of the mutation within the E1, E2, or E3 subunits is not the sole determinant of clinical outcome [11]. The nature of the variant, such as, whether it is missense, nonsense, or a deletion, also plays a role in dictating the residual enzymatic activity and overall disease manifestation. E2 and E3 [dihydrolipoamide dehydrogenase (DLD)] subunit deficiency is less common but often involves broader systemic involvement, with DLD shared by multiple mitochondrial enzyme complexes and contributing to greater metabolic instability. In our three case reports, each of the patients carried a distinct genotype—two males with a PDHA1 mutation, albeit of different types, and one female with a DLD variant, illustrating a spectrum of clinical expression and treatment response in PDCD. It is difficult to carry out definitive results of the case reports that we described correlating genotype to treatment response. In comparing the vignettes, we observe that two were of male sex and with missense mutations on the PDC E1 alpha subunit. Albeit the remaining characteristics of their mutations differed; they displayed extreme differences in illness onset and severity from one another. In *Case B* the initial manifestation was exercise-induced dystonia materializing in childhood. A resemblant pattern was documented in the work of Castiglioni et al., [84] describing a 17-year-old female with episodic dystonia. Similar to *Case B*, she too displayed FLAIR sequencing in neuroimaging that appeared at 16 years. Interestingly, her symptoms have completely remitted after chronic treatment with high doses of thiamine [84]. In *Case A*, the dearth of diagnostic confirmation resulted in suboptimal patient management, during which he received continuous intravenous glucose. While the patient also received phenylbutyrate sodium, as indicated for his hyperammonemia, and thiamine, they seem to have had no notable positive outcomes, as the perpetual glycolysis activation may have overbearingly exacerbated his state. The notion of glucose-loading among PDCD patients may lead to the aggravation of acidosis due to the blockade of the PDC, and thus its conversion to lactate. However, in this regard, the lowest possible dose of glucose administration is warranted in order to prevent hypoglycemia. With respect to the patient in *Case C*, the foreknowledge that she would be born with PDCD allowed for proper surveillance and treatment planning, despite the severity of her illness.

In this context, we highlight the importance of timely diagnosis of inborn errors of metabolism (IEM) in general. Given the reports showing that most IEM erupt shortly after birth [85], early definitive confirmation is critical in order to optimize disease surveillance, management and outcomes [86]. Though each IEM is rare unto itself, epidemiological reports show a prevalence of 2–3% of the global population [87], incurring immense strains on patients, their families, and the healthcare system [88]. Resources in clinical practice are accessible for identifying patients with metabolic diseases (i.e., hyperlactatemia with no detectable source of hypoxia) [89]; however, the process of diagnostically deciphering one IEM from another is where barriers often lie in medical institutions (high costs, inaccessible laboratory testing, and more). As demonstrated in the differential diagnoses of *Case A*, two IEM, on antithetical metabolic pathways (PDCD vs. pyruvate carboxylase deficiency), may clinically manifest indistinguishably, but require diametrically opposing dietary regimens. In this regard, recently proposed strategies for expanded newborn screening, using techniques of untargeted metabolomic profiling [90] have displayed significantly heightened detection of IEM [91]. Such an approach may be considered when primary newborn screening for metabolic diseases comes back normal.

The present study collectively reviewed treatment outcomes for PDCD including preclinical, clinical, and investigational treatments. However, we did not perform a systematic review or meta-analysis. We did not analyze treatment efficacy by variant or sex, as the available data are limited, heterogeneous, and not sufficiently powered to draw reliable conclusions. We also did not examine the safety, tolerability (adverse effects), dosing, or administration routes of the therapeutic interventions, as our goal was to provide a general descriptive overview of treatments and their effectiveness. In two instances, we observed that our focus overlapped with aspects of the study that were not originally investigated. These were: (i) in regard to DCA’s potentially neurotoxic side effects, and (ii) in reference to KD ratios as well as age of diet commencement. Multiple studies have suggested that DCA has toxic properties [92,93,94], with DCA treatment being associated with the onset or worsening of peripheral neuropathy. While DCA acts as a metabolic modulator, its long-term use has been reported to cause reversible peripheral neuropathy, particularly among adult patients [95,96,97]. DCA’s neurotoxicity may be associated with inhibition of glutathione transferase zeta-1 (GSTZ1), leading to accumulation of maleylacetoacetate and δ-aminolevulinic acid, which disrupt neuronal metabolism and promote oxidative stress [98]. Importantly, however, these findings showed that DCA’s metabolism is genotype- and age-dependent: children, especially those carrying the rapid-metabolizer GSTZ1-EGT haplotype, exhibit fewer adverse effects than adults. In adults, DCA is cleared more slowly, and higher cumulative GSTZ1 inactivation further elevates the risk of neuropathy. Thiamine supplementation was shown to ameliorate or prevent neurotoxicity, supporting a genotype- and age-adjusted dosing strategy for improved tolerability [98]. Of particular relevance, Abdelmalak et al. [99] prospectively investigated the chronic safety profile of DCA treatment among an eight-patient population of PDCD. Their findings show correlation between the PDCD genotype and suitability of DCA treatment. DCA was found to be overall well-tolerated with no notable reported incidence of peripheral nerve toxicity, in contrast to prior reports. Recent evidence on the classic KD indicates that while it is generally well-tolerated, mild-to-moderate adverse effects are common and require monitoring. A recent systematic review of 26 clinical trials of the KD among patients with epilepsy [100] found gastrointestinal disturbances (such as diarrhea or constipation), dyslipidemia, lethargy, and anorexia to be the most frequent short-term complications associated with the KD. Long-term effects include persistent gastrointestinal issues, dyslipidemia, reduced bone mineral density, renal calculi, and, less frequently, infection and hyperuricemia [100]. Furthermore, macronutrient deficiencies are to be expected. These adverse effects are typically manageable through dietary adjustments, carnitine supplementation, and modified fat-to-carbohydrate ratios, without compromising seizure or metabolic control.

In regard to the dosing of the interventions described, available evidence remains limited and mostly investigational. Thiamine has been administered at doses of 300–1000 mg/day, and riboflavin at 220–400 mg/day [11]. For DCA, twice-daily dosing of 12 mg/kg in EGT carriers and 6 mg/kg in noncarriers has been administered [101]. KD protocols may vary from high fat ratios of 3:1–4:1, to lower ratios of 1:1–2:1 considered in cases of intolerance or ketoacidosis [21]. Additionally, we did not scrutinize treatment modalities in the context of precise genetic variant type (i.e., frameshift, nonsense, or missense) or subunit location but rather grouped all genotypes of PDCD together. While all PDCD variants encumber overall enzymatic function, not all therapies are suitable for all illness forms. Furthermore, select phenotypes of PDCD are reported to cause more severe morbidity [10,11]; this detail, in addition to the unexplained heterogeneity of illness presentation, may plausibly attribute to patient outcomes more so than the administered treatment methods. Therefore, it is imperative to acknowledge that, given the small cohort sizes, retrospective study designs, and marked genetic heterogeneity of the included populations, outcomes regarding the reported treatment remain difficult to generalize.

Translating preclinical findings into clinical practice for PDCD poses substantial challenges. As a rare and genetically heterogeneous disorder, the feasibility of large-scale RCTs is notably challenging. Most evidence derives from small, genotype-diverse cohorts. This inherent variability complicates the ability to draw definitive conclusions about treatment efficacy or prognosis. Nonetheless, it remains crucial to investigate all plausible therapeutic interventions among available patient populations, as these studies provide essential insights into disease behavior, treatment response, and long-term outcomes.

This review was designed as a focused narrative synthesis of the available literature addressing the effectiveness of treatment strategies in PDCD. Relevant publications were identified through a structured search and screening process, and eligible studies were included to assess their therapeutic effectiveness. Nevertheless, the findings are limited by small cohorts, retrospective study designs, and heterogeneity within the available literature. The illustrative case series presented here intends to complement these themes by reflecting the diversity of presentation, management, disease trajectory, and treatment response as described in the literature.

## 6. Conclusions

PDCD is a rare inborn error in carbohydrate metabolism with no curative treatment to date. Integration of untargeted metabolomic profiling and expanded newborn screening panels, when clinically necessary, may facilitate the timely detection of PDCD and differentiation from other causes of CLA. Early diagnosis is critical as existing treatment methods may offer improved disease outcomes, including bettered neuropathology, reduced blood lactate levels, increased enzymatic activity, and improved prognosis. Further awareness, preclinical, and clinical trials for existing and novel treatments are necessary to improve management and outcomes and a further clinical and literature-based understanding of this illness.

## Figures and Tables

**Table 1:** Studies evaluating biochemical and clinical outcomes of therapeutic interventions among patients with PDC deficiency. CLA = congenital lactic acidosis; CSF = cerebral spinal fluid; MRI = magnetic resonance imaging; PDCD = pyruvate dehydrogenase complex deficiency; QOL = quality of life.

Treatment	Reference	Population (N)	Study Design	Duration of Treatment	Measured Outcomes	Biochemical Outcomes	Clinical Outcomes
Dichloroacetate	[29]	CLA: 53 PDCD: 17 Subunit mutations: not specified	Retrospective	11 months (average)	Biochemical response: urine/CSF/blood lactate levels Clinical response: general physical status or neurological condition	Decrease in blood/CSF lactate of at least 20% from the pretreatment lactate level among 11 PDC deficiency patients	Improved general physical status and neurological condition among three PDCD patients
	[58]	CLA: 43 PDCD: 11 Subunit mutations: not specified	Prospective, randomized, double-blind, placebo- controlled trial	3.25 years (average)	Biochemical response: blood lactate concentration in the fasted state and after a carbohydrate meal. Clinical response: neurobehavioral, neurophysiological evaluation, nerve conduction, and QOL	Decreased blood lactate caused by carbohydrate feeding (*p* < 0.001)	Trends toward improvement in clinical symptoms reported
	[34]	46 Subunit mutations: E1 alpha: 22 E1 beta: 1 Other/unknown: 23	Review of the literature, retrospective	16 months (median) (Range: 10 days to 9 years 9 months)	Biochemical response: blood and CSF lactate concentrations	Decrease in blood (*p* = 0.001) and CSF (*p* = 0.005) lactate concentrations.	None statistically analyzed. Descriptive reports of improved neuropathological symptoms
	[59]	CLA: 36 PDCD: 10 Subunit mutations: not specified	Randomized control trial, followed by an open-label study	2.38 years (median) (Range: 0.00–9.67 years)	Biochemical response: blood lactate levels; developmental outcome; survival age	Decline in both basal and meal-stimulated lactate concentrations	Suggested improved survival analysis. Worsened neuropathology
Ketogenic Diet	[60]	7 Subunit mutations: E1 alpha: 7	Prospective	Range: 1–15 years	Biochemical response: blood lactate levels Clinical response: lifespan, developmental outcomes	Lowering of blood lactate	Increased longevity and improved mental Development among three patients
	[57]	18 Subunit mutations: E1: 18 (alpha or beta not specified)	Review of the literature; retrospective	64 ± 5 months (average)	Biochemical response: Blood lactate concentration Clinical response: Neuro-developmental outcomes	No significant correlation between fat intake and blood lactate concentration	Improved neurodevelopment
	[21]	19 Subunit mutations: E1 alpha: 17 E3: 2	Longitudinal cohort study	2.9 years (median)	Biochemical response: Blood lactate concentration Clinical response: Neurocognitive and motor development; hospitalization frequency	Decreased blood lactate as compared to baseline (*p* = 0.01)	Positive effect on epilepsy, ataxia, sleep disturbance, speech/language development, social functioning, and frequency of hospitalizations
	[61]	16 Subunit mutations: E3: 16	Longitudinal cohort with matched retrospective Cohort	Individually varied	Biochemical response: blood lactate to pyruvate levels Clinical response: age of survival, MRI, yearly hospitalizations, QOL	No improvement in blood lactate to pyruvate levels	Improved survival, reduced hospitalizations, no significant improvement in QOL or MRI
Thiamine	[56]	CLA: 13 PDCD: 7 Subunit mutations: Not specified	Prospective	Individually varied	Biochemical response: blood and CSF lactate levels, PDC activity Clinical response: manifestations of clinical features	Increased PDC activity. Reduced blood and CSF lactate concentrations	Improved hypotonia, ataxia, paralysis, respiration, vomiting, and muscle pain
	[62]	19 Subunit mutations: E1 alpha: 19	Retrospective- observational	Individually varied	Clinical response: MRI, neurological, and non-neurological symptoms	None reported	Resolved brain lesions, improved neuropsychological performance, mobility, respiration, strength, energy level, development, intellectual abilities, and communication

**Table 2:** Outcomes of preclinical trials for treatments of pyruvate dehydrogenase complex deficiency. DCA = Dichloroacetate; PDC = pyruvate dehydrogenase complex.

Treatment		Reference	Design/Model System	Outcome Summary
**Dichloroacetate**		[32]	In vitro: fibroblast human cells PDCD vs. control sample	Increase in maximal PDC activity in PDC-deficient cell lines showing reduced immunoreactive E1
		[63]	In vitro: human fibroblasts and lymphocytes	Mean increase in PDC activity by 125% and 70% among two samples
**Ketogenic diet**		[64]	In vivo: zebrafish model; rat model	Restored vision, promoted feeding behavior, reduced lactic acidosis, and increased survival.
**Phenylbutyrate**		[65]	In vivo; in vitro unphosphorylated PDH-E1 alpha and PDC enzyme activity	Increased unphosphorylated PDC-E1alpha and PDC enzyme activity in mouse brain, muscle, and liver
**Gene Therapy**		[53]	Recombinant adeno-associated virus 2 vectors In vivo: rat model In vitro: human fibroblasts	Stable gene transfer, expression, and translocation; ~30% restored PDC activity
**Combination Therapies**	**Gene Therapy and Dichloroacetate**	[66]	In vitro: self-complementary adeno-associated virus serotype-specific vectors and dichloroacetate	Gene vectors increased E1 alpha expression 40–60%; DCA increased PDC activity up to 90% activity and expression of E1 alpha protein
	**Phenylbutyrate and Dichloroacetate**	[65]	In vivo: mouse model In vitro: human control fibroblasts	Greater increase in PDC activity compared to each drug alone

**Table 3:** Arterial blood gas trends Case A. Ammonia and blood gas trends of male infant described in *Case A*, tested over a three-day span at different time points from hospital admission until demise. Point 1 marks the commencement of treatment with bicarbonate and dextrose infusion. Normal ranges: pH: 7.35–7.45, bicarbonate (HCO_3_): 22–26 mmol/L, lactate: 0.5–2.2 mmol/L, partial pressure of carbon dioxide (pCO_2_): 35–45 mmHg.

Time Point	pH	HCO_3_ (mmol\L)	Lactate (mmol\L)	pCO_2_ (mmHg)	Ammonia (µg/dL)
0—Admission	7.27	9.6	10.5	21	Not tested
1	7.26	9	10.5	20	Not tested
2	7.16	15.7	10	44	74.3
3	7.13	7	10	28	97.2
4	7.09	5.7	18.1	23	221.7
5	6.9	3.6	18.1	29	370.4
6	6.82	8.3	18.1	22	383.1
7—Death	6.8	0	24.8	36	1170.9

**Table 4:** Feature comparison of case series. Abbreviations: DLD, dihydrolipoamide dehydrogenase; DWI, diffusion-weighted imaging; FLAIR, fluid-attenuated inversion recovery; FTT, failure to thrive; IUGR, intrauterine growth restriction; IV, intravenous; KD, ketogenic diet; MRI, magnetic resonance imaging; PEG, percutaneous endoscopic gastrostomy.

	Case A	Case B	Case C
Sex	Male	Male	Female
Genotype Variant	PDHA1 c.763C>A p.(Pro255Thr), Missense, de novo	PDHA1 c.376C>T p.(Arg126Cys), Missense, hemizygous	DLD (E3) D479V, Missense, homozygous, familial
Age at Presentation	Neonatal onset, metabolic crisis at 1 month	3 years: dystonia 7 years: metabolic crisis triggered by infection	Neonatal onset
Birth Events, Growth	37 weeks, 2170 g, IUGR	Term, uneventful birth and growth	Term, 2480 g, siblings deceased from DLD
Major Clinical Features	Hypotonia, hypothermia, feeding difficulty, lactic acidosis, hyperammonemia, inguinal hernia	Exercise-induced dystonia, nystagmus, asymmetric smile, toe-walking, metabolic crises triggered by infection	Neonatal lactic acidosis, hypoglycemia, developmental delay, FTT, severe disability, recurrent metabolic crises
Neuroimaging	Postmortem MRI: mild ventricular bleed, thalamic infarct	MRI FLAIR: globus pallidus hyperintensity, DWI restriction	MRI at 1 year: bilateral globus pallidus DWI restriction, thin corpus callosum, ventriculomegaly, extraxial space enlargement
Treatment	Acute: IV dextrose, arginine, dopamine, vitamins B1/B2/B7/B12, carnitine, sodium bicarbonate, phenylbutyrate, sodium benzoate, antibiotics	Chronic: KD, trihexyphenidyl, clobazam, L-carnitine, tetrabenazine, pyridoxine, vitamin B12, thiamine	Acute: Neonatal KD via PEG, IV sodium bicarbonate, dextrose, intralipid Chronic: KetoCal via PEG, sodium bicarbonate, K-citrate, levetiracetam
Outcome	Died at 1 month (lactic acidosis, hyperammonemia)	Age 14 years: mitochondrial dystonia, impaired ambulation	Age 5 years: severe developmental delay, nonverbal, FTT, frequent metabolic crises

## Data Availability

Data sharing is not applicable to this article as no datasets were generated or analyzed during the current study.

## Abbreviations

The following abbreviations are used in this manuscript:

ATP	Adenosine triphosphate
CLA	Congenital lactic acidosis
CSF	Cerebrospinal fluid
DCA	Dichloroacetate
DLD	Dihydrolipoamide dehydrogenase
DWI	Diffusion-weighted imaging
FDA	Food and Drug Administration
FLAIR	Fluid-attenuated inversion recovery
GSTZ1	Glutathione transferase zeta-1
IEM	Inborn errors of metabolism
IUGR	Intrauterine growth restriction
KD	Ketogenic diet
LDH	Lactate dehydrogenase
MRI	Magnetic resonance imaging
NAC	N-acetylcysteine
PDC	Pyruvate dehydrogenase complex
PDCD	Pyruvate dehydrogenase complex deficiency
PDHA1	Pyruvate dehydrogenase E1 alpha subunit gene
PDK	Pyruvate dehydrogenase kinase
PDP	Pyruvate dehydrogenase phosphatase
PEG	Percutaneous endoscopic gastrostomy
PRISMA	Preferred Reporting Items for Systematic Reviews and Meta-Analyses
RCT	Randomized controlled trial
rAAV	Recombinant adeno-associated virus
ROS	Reactive oxygen species
SD	Standard deviation
SUMC	Soroka University Medical Center
TCA	Tricarboxylic acid
